# Study of Plasma Biochemistry and Plasma Metabolomics Differences in Montbéliard and Holstein Backcross and Holstein Heifers

**DOI:** 10.3390/ani14162294

**Published:** 2024-08-06

**Authors:** Haihui Wang, Haomiao Chang, Hantong Weng, Yunfei Zhai, Hanfang Zeng, Shujie Li, Zhaoyu Han

**Affiliations:** College of Animal Science and Technology, Nanjing Agricultural University, Nanjing 210095, China; 2022105001@stu.njau.edu.cn (H.W.); 15117424@njau.edu.cn (H.C.); 2022105011@stu.njau.edu.cn (H.W.); kint214@163.com (Y.Z.); 2020205004@stu.njau.edu.cn (H.Z.); lishujie@njau.edu.cn (S.L.)

**Keywords:** antioxidant index, immune index, plasma metabolomics

## Abstract

**Simple Summary:**

In this study, we analyzed the plasma biochemistry and plasma metabolome of 12-month-old Montbéliard and Holstein backcross and purebred Holstein heifers, with the aim of determining differences in the performance of these two breeds. The findings revealed that the backcrossed heifers are superior to Holsteins with respect to certain antioxidant indices and immune performance. We detected a total of 30 metabolites showing significantly differential levels between the two groups, of which 17 and 13 were significantly up- and down-regulated. Collectively, our findings in this study indicate that compared with 12-month-old purebred Holstein heifers, Montbéliard and Holstein backcross heifers of the same age are characterized by higher antioxidant capacity and immunity.

**Abstract:**

Holstein cattle are the main breed of dairy cattle in China. However, given the high degree of purebred selection of Holstein cattle, Chinese dairy cattle are increasingly being characterized by poor disease resistance, poor quality, and declining fertility. In this study, using Montbéliard × Holstein cattle as females and Montbéliard bulls as males for backcross breeding, we sought to provide a reference for improving the quality and performance of Holstein cattle and enhancing the efficiency of dairy farming. On the basis of similar physiological status and age, we selected 24 Montbéliard and Holstein backcross heifers and 11 Holstein heifers fed the same formula for comparative analyses. Plasma samples collected for plasma biochemical index analyses revealed that the content of ALB and BUN in the Montbéliard and Holstein backcross heifers was 20.83% (31.62 g/L to 26.17 g/L) and 42.36% (6.89 mmol/L to 4.84 mmol/L) higher than in the Holsteins (*p* < 0.01). The ALB/GLB (0.90 to 0.60, *p* < 0.05) was significantly higher in Montbéliard and Holstein backcross heifers than in Holstein heifers. Similarly, the activity of CAT in the backcross heifers was 61.28% (4.29 U/mL to 2.66 U/mL) higher than that in the Holstein heifers (*p* < 0.05). Although the activity of GSH-Px in the backcross heifers also showed an increasing trend, the difference did not reach the level of statistical significance (*p* = 0.052). Compared with Holstein heifers, the concentrations of IgA, IgG, and IL-4 were elevated by 32.52% (24.90 μg/mL to 18.79 μg/mL, *p* < 0.01), 13.46% (234.32 μg/mL to 206.53 μg/mL, *p* < 0.01), and 14.59% (306.27 pg/mL to 267.28 pg/mL, *p* < 0.05), and the contents of IL-6 and TNF-α were decreased by 15.92% (215.71 pg/mL to 256.55 pg/mL, *p* < 0.01) and 32.17% (7.17 ng/mL to 10.57 ng/mL, *p* < 0.01) in the plasma of Montbéliard and Holstein backcross heifers. Among the experimental heifers, five animals from each of the two groups were selected for plasma metabolomic analysis based on untargeted liquid chromatography–mass spectrometry. A comparison of the differential metabolites between the two heifer breeds revealed an up-regulation of d-glucuronic acid, s-glutathionyl-l-cysteine, and oleic acid levels in the backcross cattle compared with those in the Holstein heifers. We speculate that changes in the levels of these metabolites may be associated with an enhancement of the anti-inflammatory, antioxidant, and immune systems in these backcross heifers. Collectively, our findings in this study indicate that compared with 12-month-old purebred Holstein heifers, Montbéliard and Holstein backcross heifers of the same age are characterized by higher antioxidant capacity and immunity.

## 1. Introduction

During the course of Holstein cattle breeding, genetic selection has contributed to improving their production performance, and these cattle are noted for their superior milk production performance and adaptability, making Holstein cattle a major dairy breed [1]: approximately 95% of the dairy cattle breeds in China are of the Holstein milk cow variety [2]. However, in recent years, with the continued improvement of Holstein production levels and high levels of purebred breeding, there have been corresponding negative consequences, including reduced service life, poor quality, and a decline in health and reproductive capacity [3], thereby contributing to reductions in the production performance of these dairy cattle, which has accordingly raised concerns among dairy farmers and dairy cattle breeding experts [4,5]. In this regard, reproductive and disease resistance traits have been established to be low-heritability traits, which can, nevertheless, be improved through crossbreeding [5]. Consequently, the crossbreeding of Holstein cattle is being actively studied worldwide with a view toward effectively resolving the declining performance and characteristics of these cattle. An increasing number of crossbreeding studies are accordingly using Holstein cattle to crossbreed with a range of other cattle breeds, the findings of which tend to indicate that compared with purebred Holsteins, crossbred cattle have a number of distinct advantages in terms of fertility, growth performance, and disease resistance, including Simmental [6], Fleckvieh [7], Normande and Scandinavian Red [8], Brown Swiss [9], and Montbéliard [6] cattle. Among breeds, the Montbéliard (also referred to as Simmental cattle) is a renowned meat cattle breed from France, which is the second largest breed of dairy cattle in France, derived via the long-term selection of the Swiss Pie Rouge breed. Montbéliard cattle are tall, with small heads, wide round hips, and relatively well-developed thigh muscles [10]. The adult cows weigh approximately 650–750 kg, whereas bulls can weigh up to 800–1000 kg [11]. These cattle are characterized by high adaptability and disease resistance, tolerance to roughage, high reproductive rate, long service life [12], good breast structure, rapid milk discharge, and good lactation, and they produce milk with high fat and protein contents [13]. Experimental crossbreeding between Montbéliard and Holstein cattle indicated that body weight, hip height, chest circumference, tube circumference, body height, and head length of the 4-month-old hybrid calf were 15.07 kg, 5.36 cm, 5.77 cm, 0.82 cm, 3.26 cm, and 1.67 cm higher than those of the pure Holsteins, respectively (*p* < 0.05). The results have revealed that the Montbéliard × Holstein offspring are characterized by a superior growth trend [14]. Moreover, these crossbreds produce milk with significantly higher fat and protein percentages, and the birth month and empty days are significantly lower than those of Holstein cattle [5].

Metabolomics is a branch of system biology that seeks to explain pathological and physiological conditions based on the detection and quantification of metabolites [15]. It is typically performed using one of two main approaches, namely, targeted and untargeted metabolomics [16]. At present, the most widely used metabolomics research methods are NMR, MS, LC–MS, GC–MS, and CE–MS [17]. The application of metabolomics in the field of ruminant research includes studies of the metabolomes of the rumen [18], liver [19], feces [20], urine [21], breast [22], and blood [23,24]. Throughout the body, blood is distributed within organs and tissues, in which its constituents are involved in a diverse range of metabolic processes. Accordingly, analyses of the blood metabolomics can contribute to determining differences in the metabolite profiles of different breeds of animals, and thereby facilitate assessments of breed-related differences in production performance and metabolic characteristics. For example, Karisa [25] used NMR to study the blood metabolomics of beef cattle, screening differential metabolites related to production performance, such as carnitine, creatine, and urate, for application in beef cattle breeding. Similarly, using LC-MS/MS and NMR, Foroutan [26] detected metabolites such as leucine, formate, and lysophosphatidylcholine as differential metabolic markers in the serum metabolomics of cattle, and successfully identified important biomarkers for classifying and predicting feed utilization efficiency. Ilve [27] used MS to analyze Holstein cattle, and the results showed that the largest change in plasma metabolites in early lactation was related to the level of unsaturated fatty acids. Consequently, screening differential blood metabolites and gaining an understanding of their metabolic functions can contribute to evaluating metabolic differences among different varieties.

In this study, we analyzed the plasma biochemistry and metabolomics of 12-month-old Montbéliard and Holstein backcross and purebred Holstein heifers, with the aim of determining differences in the performance of these two breeds. We hypothesized that by analyzing plasma biochemical indices and differential plasma metabolites of the two breeds, it would be possible to establish that certain performance traits of the backcross heifers are superior to those of Holsteins, thus highlighting the hybrid benefit of Montbéliard and Holstein cattle, and providing data that can contribute to improving local dairy breeds and thereby enhance the overall benefits to dairy farming.

## 2. Materials and Methods

### 2.1. Animal Experiments

For the purposes of this study, we selected heifers born on a cattle farm in Xuzhou City, Jiangsu Province, China, from October to December 2021. All heifers were free of malformations or any history of previous diseases, had been vaccinated, and only animals deemed to be healthy were utilized. All test heifers (386.47 ± 20.35 kg body weight) were in a non-estrus state. The study was approved by the Experimental Animal Welfare and Ethics Committee of the Nanjing Agricultural University (Approval Code: SYXK(Su)2017-0027; approval date: 7 December 2017). On the basis of a similar age, we selected 24 twelve-month-old Montbéliard and Holstein backcross heifers obtained by backcrossing Montbéliard × Holstein cattle as dams and French Montbéliard cattle as sires as an experimental group. As controls, we used 11 purebred Holstein heifers born on the farm at the same time. Heifers in both the experimental group and the control group were maintained under the same feeding and management conditions (Table 1), during which they were fed in a scatterbox style with free access to food and water. 

### 2.2. Sample Collection and Determination

At 1 to 2 h after morning feeding, we used disposable blood samplers to collect 10 mL samples of blood from the caudal vein of heifers, which were transferred to anticoagulation tubes. Having left the samples to stand for 1 h, the plasma was separated by centrifuging at 3500 r/min for 10 min and thereafter transferred to 1.5 mL centrifuge tubes and stored at −20 °C for subsequent analyses of plasma biochemical and hormone levels. We monitored TP, ALB, BUN, GLU, ALT, AST, T-AOC, SOD, GSH-Px, MDA, and CAT using the microplate method. Respective test kits were purchased from Nanjing Jiancheng Bioengineering Institute (Nanjing, China; #A045-4, #A028-2-1, #C013-2-1, #A154-1-1, #C009-2-1, #C010-2-1, #A015-3-1, #A001-3, #A005-1, #A003-1, #A007-1-1, respectively). These were analyzed by using a Microplate Reader (Spark, TECAN, Männedorf, Switzerland). IgA, IgG, IL-4, IL-6, and TNF-α were determined by ELISA. Respective test kits were purchased from Nanjing Angle gene Biotechnology Co., Ltd. (Nanjing, China; #ANG-E61022B, #ANG-E61025B, #ANG-E61084B, #ANG-E61008B, #ANG-E61006B). These indexes were detected by using the Microplate Reader (F50, TECAN, Männedorf, Switzerland). All testing procedures are carried out in strict accordance with the manufacturer’s instructions.

### 2.3. Metabolomics Analysis

For each group, we randomly selected 5 heifers of similar physiological conditions and age, from which 5 mL blood samples were collected from the caudal vein and placed in anticoagulation tubes. The blood was immediately centrifuged at 3000 r/min for 10 min to obtain plasma, which was transferred to cryopreservation tubes and stored at −80 °C. These plasma samples were subsequently sent to PANOMIX (Suzhou, China) for blood metabolomic determinations.

#### 2.3.1. Sample Preparation for LC-MS Analysis

The blood samples were pre-processed and detected on the machine according to the requirements of liquid chromatography–mass spectrometry non-targeted metabolomics. The plasma samples were removed from −80 °C, thawed at 4 °C, vortexed for 1 min to mix evenly, and 100 μL samples were accurately transferred into 2 mL centrifuge tubes. Then, 400 μL methanol was added (stored at −20 °C), the mixture was vortexed for 1 min and centrifuged for 10 min at 12,000 rpm and 4 °C, and finally, all the supernatant was taken, transferred to 2 mL centrifuge tubes, concentrated, and dried. A total of 150 L of a 2-chloro-l-phenylalanine solution (4 ppm), prepared using 80% methanol in water, was added and stored at 4 °C to facilitate the re-dissolution of the sample. Subsequently, the supernatant was filtered through a 0.22 µm membrane into a test bottle for LC-MS metabolomics analysis [28]. 

#### 2.3.2. Liquid Chromatography Conditions

The chromatography analysis was performed on a Thermo Vanquish (Thermo Fisher Scientific, Waltham, MA, USA). Chromatography was performed on an ACQUITY UPLC HSS T3 (2.1 × 150 mm, 1.8 µm) column (Waters, Milford, MA, USA) at a flow rate of 0.25 mL/min, column temperature of 40 °C, and sample size of 2 μL. For LC-ESI (+)-MS analysis, the mobile phases were 0.1% formic acid in acetonitrile (B2) and 0.1% formic acid in water (A2). For LC-ESI (−)-MS analysis, the analysis was carried out with acetonitrile (B3) and 5 mM ammonium formate (A3) [29]. The gradient elution program of positive and negative ion modes is shown in Table 2.

#### 2.3.3. Mass Spectrum Conditions

Mass spectrometric detection of metabolites was conducted using the Orbitrap Exploris 120 (Thermo Fisher Scientific, USA) equipped with an electrospray ionization (ESI) source. The acquisition was performed in a simultaneous full MS-ddMS2 mode, which utilizes data-dependent MS/MS. The operational parameters were set as follows: sheath gas pressure at 30 arbitrary units (arb), auxiliary gas flow at 10 arb, and a spray voltage of 3.50 kV for ESI(+) and −2.50 kV for ESI(−). The capillary temperature was maintained at 325 °C, while the MS1 scan range was established at *m*/*z* 100–1000. The resolving power for MS1 was 60,000 full widths at half maximum (FWHMs), with four data-dependent scans per cycle and an MS/MS resolving power of 15,000 FWHM. The normalized collision energy was set at 30%, and the dynamic exclusion time was configured to automatic [30]. 

### 2.4. Statistical Analysis

Experimental data pertaining to plasma biochemistry were processed using Excel 2019 and were subsequently subjected to a one-way ANOVA using IBM SPSS Statistics 26.0 (SPSS Inc., Chicago, IL, USA). Data are expressed as the means and standard error, with the significance of differences being set at *p* < 0.05. The trend was considered when 0.05 < *p* < 0.1. Metabolites exhibiting VIP values greater than 1.0 and *p*-values from two-tailed Student’s *t*-tests less than 0.05 were classified as differential metabolites.

The raw mass spectrometry files were converted to the mzXML file format by MSConvert in the Proteowizard package (v3.0.8789) [31]. The RXCMS software (v3.12.0) package was utilized for peak detection, filtering, and alignment, in order to generate a quantitative list of substances [32]. The main parameters are bw = 2, ppm = 15, peakwidth = c (5, 30), mzwid = 0.015, mzdiff = 0.01, and method = “centWave”. Ropls software (v3.6.5) [33] was used for all multivariate data analyses and modeling. After scaling data, models were built on PCA, PLS-DA, and OPLS-DA. All the models evaluated were tested for overfitting with permutation tests, differential metabolite screening was performed based on OPLS-DA variables (VIP), and the *p*-value was calculated by statistical tests: VIP > 1 and *p* < 0.05. 

## 3. Results

### 3.1. Comparison of Plasma Biochemical Indicators in 12-Month-Old Montbéliard and Holstein Backcross and Holstein Heifers

As is evident from Table 3, the plasma levels of ALB and BUN in 12-month-old Montbéliard and Holstein backcross heifers were 20.83% (31.62 g/L to 26.17 g/L) and 42.36% (6.89 mmol/L to 4.84 mmol/L) higher than in the Holsteins (*p* < 0.01). The ALB/GLB values (0.90 to 0.60, *p* < 0.05) were significantly higher in Montbéliard and Holstein backcross heifers than Holstein heifers, with no significant difference in the levels of TP, GLU, ALT, and AST (*p* > 0.05).

### 3.2. Comparison of Plasma Antioxidant Function in 12-Month-Old Montbéliard and Holstein Backcross (MH) and Holstein Heifers (H)

As shown in Table 4, the activity of CAT in plasma was 61.28% (4.29 U/mL to 2.66 U/mL) higher in the 12-month-old Montbéliard and Holstein backcross heifers than in pure Holstein heifers (*p* < 0.05), whereas GSH-Px activity showed an increasing trend (*p* = 0.052). However, there were no significant differences between the two breeds regarding T-AOC, the activity of SOD, and the contents of MDA (*p* > 0.05).

### 3.3. Comparison of Plasma Immune Function in 12-Month-Old Montbéliard and Holstein Backcross (MH) and Holstein Heifers (H)

Compared with Holstein heifers, the concentrations of IgA, IgG, and IL-4 were elevated by 32.52% (24.90 μg/mL to 18.79 μg/mL, *p* < 0.01), 13.46% (234.32 μg/mL to 206.53 μg/mL, *p* < 0.01), and 14.59% (306.27 pg/mL to 267.28 pg/mL, *p* < 0.05), and the contents of IL-6 and TNF-α were decreased by 15.92% (215.71 pg/mL to 256.55 pg/mL, *p* < 0.01) and 32.17% (7.17 ng/mL to 10.57 ng/mL, *p* < 0.01) in the plasma of Montbéliard and Holstein backcross heifers (Table 5). 

### 3.4. Comparison of the Plasma Metabolomics of 12-Month-Old Montbéliard and Holstein Backcross (MH) and Holstein Heifers (H)

**Figure 1:**
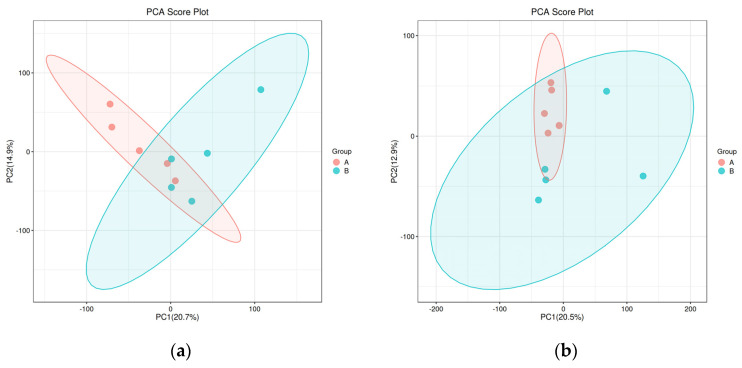
Plot of principal component analysis scores of plasma metabolites in positive (**a**) and negative (**b**) ion modes in Montbéliard and Holstein backcross heifers and Holstein heifers. Note: The R2X in positive and negative ion modes is 0.605 and 0.576, respectively. The red dots (A) represent Holstein heifers, while the blue circles (B) represent Montbéliard and Holstein backcross heifers.

**Figure 2:**
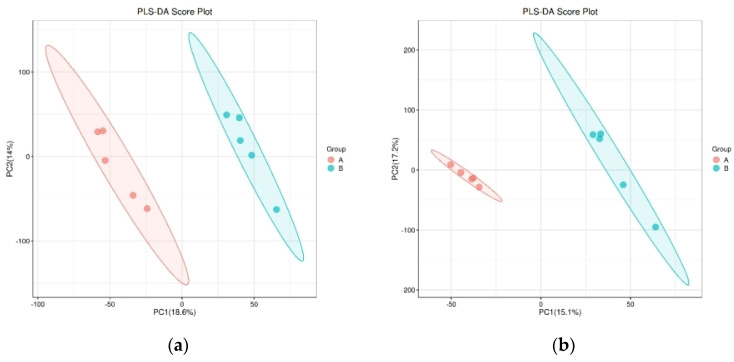
The partial least-squares discriminant analysis scatter plots between the two groups’ plasma metabolome in positive (**a**) and negative (**b**) ion modes. Note: In positive ion mode, R2Y and Q2 are 0.995 and 0.741, respectively, while in negative ion mode, R2Y and Q2 are 0.996 and 0.769, respectively.

In the PCA scores of both groups, R2X is greater than 0.5 in both positive and negative ion modes, but there is no significant separation between the two groups (Figure 1). As shown in Figure 2, PLS-DA score plots of the metabolic profiles reveal different trends between the two heifer groups. R2Y and Q2 values were greater than 0.5 in positive and negative ion modes, thereby indicating that the model of plasma metabolomics could be used to assess the differential metabolites in the control and experimental group heifers.

**Figure 3:**
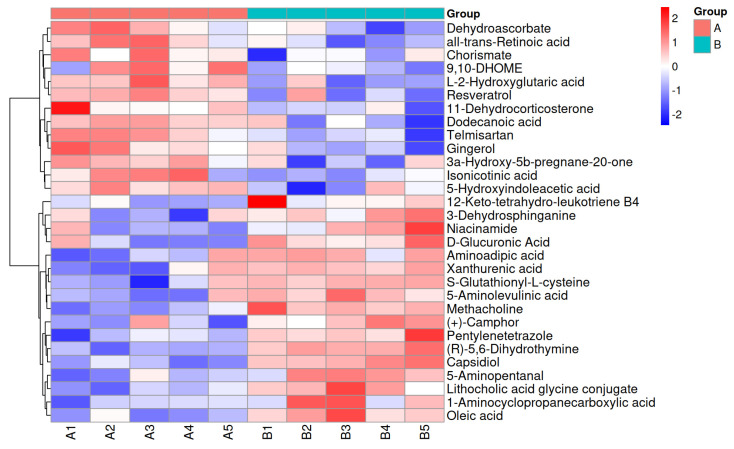
Heat maps of plasma differential metabolites in 12-month-old Montbéliard and Holstein backcross and Holstein heifers. Note: The relative content in the figure is represented by variations in color. A higher expression level is indicated by a more intense red, while a lower expression level is denoted by a deeper blue. The red (A) represent Holstein heifers, while the blue (B) represent Montbéliard and Holstein backcross heifers.

In total, we identified 30 differential plasma metabolites in the two groups based on thresholds of VIP > 1 and a *t*-test *p*-value < 0.05 in PLS-DA, among which 17 and 13 were up- and down-regulated, respectively, in the Montbéliard and Holstein backcross heifers (Figure 3).

## 4. Discussion

To a certain extent, blood biochemical indicators can reflect metabolic levels and the health status of animals [34]. TP is the sum of ALB and GLB. ALB is a kind of protein synthesized by the liver, accounting for more than 50% of the TP content, which has important functions such as participating in the transport of plasma substances, maintaining plasma osmotic pressure and coordinating protein metabolism [35]. GLB is produced by immune organs, and its content can reflect the strength of the body’s immune capacity and protein metabolism [36]. Lysine is capable of elevating plasma ALE concentration [37]. In this research, the plasma ALB of Montbéliard and Holstein backcross heifers was higher than that of Holsteins, probably because the backcross heifers were able to make better use of lysine for plasma protein synthesis. These findings are consistent with those reported previously regarding ALB contents in Holstein and Fleckvieh × Holstein F1 lactating cows, which were found to be higher than those in Holstein cows [7]. This indicates that the liver of backcross heifers might have a stronger ability to synthesize protein, and can better absorb and transport proteins produced by the body, which is conducive to the growth and development of the body. BUN, another routinely used blood indicator, is a product of protein metabolism, which is mainly derived from the rumen digestion and absorption of nitrogen, with a lower BUN content being more conducive to nitrogen deposition in the animal body. Our findings in this study revealed that compared with Holstein heifers, the sera of Montbéliard and Holstein backcross heifers contained significantly higher amounts of BUN, which might be related to dry matter intake.

It has been shown that the antioxidant capacity of animals is associated with the content of oxygen radicals in the body [38]. Under conditions of normal respiration, most of the intracellular oxygen combines with glucose and fat in organelles, which are then converted into energy for absorption and utilization by the body. However, a certain proportion of oxygen molecules undergo conversion to oxygen free radicals, which, if produced in excess, can contribute to the development of metabolic disorders. The antioxidant capacity of animals is determined by the antioxidant system, which comprises a range of enzymic and nonenzymic agents, including SOD, GSH-Px, and CAT [39]. Given their efficient elimination of oxygen radicals, GSH-Px and CAT are typically used as indicators to evaluate the antioxidant capacity of the body [40]. The antioxidant properties of glutathione are primarily accomplished by the inhibition of lipid peroxidation via glutathione peroxidase 4. Glutathione can be directly generated from cysteine or through the transsulfuration of methionine to cysteine [41]. In the present study, we detected significantly higher activity of GSH-Px in the Montbéliard and Holstein backcross heifers. This result may be due to the significant up-regulation of s-glutathionyl-l-cysteine in the plasma metabolites of the Montbéliard and Holstein backcross heifers to promote the metabolism of cysteine and methionine in the organism, which in turn improves the activity of GSH-Px in the heifers. The higher CAT activity in Montbéliard and Holstein backcross heifers than in Holstein heifers may be due to the fact that backcross heifers show higher efficiency in energy metabolism, which in turn promotes the synthesis of intracellular antioxidant enzymes and enhances their scavenging capacity for free radicals in the organism.

Blood immunoglobulins, including IgA, IgG, IgM, and IgY, can provide a direct reflection of the humoral immunity status of animals. In response to cellular attack, these immunoglobulins combine with antigens to alleviate the damage caused to cells. Among these molecules, IgA has been established to suppress viral disoperation and destroy viruses, whereas IgG, which tends to be the most abundant of the immunoglobulins, accounting for approximately 75% of the total content, plays a key role in humoral immunity [42]. Our findings in the present study revealed that compared with those in Holstein heifers, the contents of IgA and IgG in 12-month-old Montbéliard and Holstein backcross heifers were significantly higher. The reason for this result may be that rumen microorganisms of Montbéliard and Holstein backcross heifers have a strong ability to utilize butyric acid, which can promote the proliferation and differentiation of immune cells and improve the immune function of the body. Contrastingly, in a previous study comparing 5-month-old German Simmental × Holstein and Holstein cattle, the authors detected no significant differences between these cattle with respect to serum IgA and IgG levels, and it was thus assumed that these two breeds were characterized by similar levels of immunity [43]. We speculate that the findings of these differences may be attributed to differences in animal age and diet management between the two studies, and differences in feeding management between different cattle may lead to different nutrient intake, which in turn affects blood immunoglobulin levels. 

In response to pathogen invasion, immune-related cells secrete polypeptides that combine with receptors on T and B cells to regulate immunity [44]. In this context, the cytokine IL-4, produced by activated T cells, promotes T-cell differentiation and the production of IgE by these cells [45]. The levels of another interleukin, IL-6, are closely associated with inflammation and the development of autoimmune diseases, and it has been established to regulate Th 17 cell differentiation. On the other hand, TNF-α, which is secreted by macrophages and monocytes, functions as a pro-inflammatory factor that accelerates cell proliferation and apoptosis [46]. It has been suggested that oleic acid reduces the expression of pro-inflammatory factors TNF-α, IL-6, and IFN-γ [47]. In the present experiment, the contents of TNF-α and IL-6 were significantly lower in Montbéliard and Holstein backcross heifers than in Holsteins. This might be attributed to the significant up-regulation of oleic acid in the plasma metabolites of backcross heifers, which had suppressed expression of TNF-α and IL-6 and decreased contents of both in the plasma. TCR signals promote the expression of IL-4 by mediating NFAT protein [48]. IL-4 mainly stimulates the production of antibodies by activating B cells. 

d-glucuronic acid is an important intermediary metabolite generated in the synthesis of glucuralactone and hyaluronic acid, which is primarily involved in the metabolism of nucleotides, proteins, and bile salts, and has been established to have certain anti-inflammatory, cytoprotective, and immunomodulatory effects [49]. d-glucuronic acid is widespread in animals, and it can stimulate TH1-dependent immune responses by increasing the number of antibodies in the blood [50]. It can undergo conversion to ascorbic acid via a series of enzymatic reactions and thereby contribute to the suppression of inflammatory responses [49]. d-glucuronic acid serves as the precursor for the synthesis of l-ascorbic acid in mammals. d-glucuronic acid is reduced by NADPH to yield l-gulonic acid, and l-gulonic lactone is formed under the influence of lactase, subsequently followed by oxidation to generate l-ascorbic acid [51]. l-ascorbic acid protects cells by restricting ROS accumulation through its oxidative attributes. The plasma metabolite d-glucuronic acid was significantly increased in Montbéliard and Holstein backcross heifers in this experiment. The result may be that the glucose metabolism in backcross heifers promotes the metabolism of ascorbic acid and uronic acid, thus improving the immune function of heifers and anti-inflammatory and antioxidant abilities.

s-glutathionyl-l-cysteine is a carboxylic acid derivative, which is the outcome of the glutathionylation of cysteine [52]. The function of s-glutathionylation within the body mainly lies in transducing redox signals and preventing irreversible oxidation of cysteine [53]. Glutathione is a tripeptide composed of glutamic acid, cysteine, and glycine, which plays a crucial role in the body’s antioxidant mechanism. s-glutathionyl-l-cysteine is enriched in cysteine and methionine metabolic pathways and can promote cysteine and methionine metabolism in the organism. Cysteine and methionine play important roles as sulfur amino acids in organismal metabolism, immunity, and oxidation [54]. The monounsaturated fatty acid oleic acid has been demonstrated to play key roles in autoimmune diseases [55], contributes to enhancing immune system function [56], and has antioxidant properties [57]. Oleic acid is an immunomodulator with anti-inflammatory effects, which plays a beneficial anti-inflammatory role by regulating microRNA expression [58]. Oleic acid can exert anti-inflammatory effects by inhibiting pro-inflammatory factors such as MAPKs, Cox-2, and NF-kappa B [59]. Oleic acid is enriched in saturated fatty acid biosynthesis and unsaturated fatty acid biosynthesis metabolic pathways, which may influence nutrient uptake in dairy cows by affecting fatty acid metabolic pathways, thereby improving body resistance to oxygen.

## 5. Conclusions

In summary, ALB, GSH-Px, CAT, IgA, IgG, and IL-4 were increased, IL-6 and TNF-α were decreased, and plasma differential metabolites with antioxidant and anti-inflammatory effects were significantly increased in 12-month-old Montbéliard and Holstein backcross heifers.

## Figures and Tables

**Table 1:** Ingredients and chemical composition of diets.

Ingredient	Content, % of Diets	Chemical Composition, % of DM	Content, % of DM
Wheat straw	6	CP	18.59
Straw	26	NDF	32.93
Integrated feed	10.4	ADF	17.62
Concentrate Supplement	1.6	EE	4.77
Brewers’ grains	12	Ash	9.34
Maize silage	44	Ca	0.92
Total	100	P	0.78

**Table 2:** A and B are positive and negative ion mode elution procedures, respectively.

A		B	
Time/min	Mobile Phase B2/%	Time/min	Mobile Phase B3/%
0–1	2	0–1	2
1–9	2–50	1–9	2–50
9–12	50–98	9–12	50–98
12–13.5	98	12–13.5	98
13.5–14	98–2	13.5–14	98–2
14–20	2	14–17	2

**Table 3:** Comparison of plasma biochemical indicators in 12-month-old Montbéliard and Holstein backcross (MH) and Holstein heifers (H).

Items	H	MH	*p*-Value
TP, g/L	72.50 ± 3.84	71.00 ± 1.92	0.699
ALB, g/L	26.17 ± 1.04	31.62 ± 0.81	<0.001
GLB, g/L	46.34 ± 4.07	39.38 ± 2.41	0.131
ALB/GLB	0.60 ± 0.05	0.90 ± 0.08	0.019
BUN, mmol/L	4.84 ± 0.25	6.89 ± 0.33	<0.001
GLU, mmol/L	3.69 ± 0.14	3.91 ± 0.11	0.271
ALT, U/L	6.62 ± 0.58	7.12 ± 0.52	0.563
AST, U/L	14.96 ± 1.79	17.21 ± 0.86	0.206

**Table 4:** Comparison of plasma antioxidant function in 12-month-old Montbéliard and Holstein backcross (MH) and Holstein heifers (H).

Items	H	MH	*p*-Value
T-AOC, mmol/L	0.31 ± 0.02	0.36 ± 0.03	0.210
SOD, U/L	17.90 ± 0.51	18.95 ± 0.45	0.167
GSH-Px, μmol/L	107.90 ± 11.84	145.17 ± 11.24	0.052
MDA, nmol/mL	2.73 ± 0.35	3.01 ± 0.37	0.646
CAT, U/mL	2.66 ± 0.30	4.29 ± 0.46	0.029

**Table 5:** Comparison of plasma immune function in 12-month-old Montbéliard and Holstein backcross and Holstein heifers.

Items	H	MH	*p*-Value
IgA, μg/mL	18.79 ± 0.61	24.90 ± 0.73	<0.001
IgG, μg/mL	206.53 ± 6.90	234.32 ± 5.79	0.008
IL-4, pg/mL	267.28 ± 11.32	306.27 ± 8.72	0.014
IL-6, pg/mL	256.55 ± 6.86	215.71 ± 4.47	<0.001
TNF-α, ng/mL	10.57 ± 0.36	7.17 ± 0.32	<0.001

## Data Availability

The original contributions presented in the study are included in the article, further inquiries can be directed to the corresponding authors.

## Abbreviations

Item	Unit
NMR	nuclear magnetic resonance
MS	mass spectrometry
LC–MS	liquid chromatography–mass spectrometry
GC–MS	gas chromatography–mass spectrometry
CE–MS	capillary electrophoresis–mass spectrometry
CP	crude protein
NDF	neutral detergent fiber
ADF	acid detergent fiber
EE	ether extract
Ca	calcium
P	phosphorus
DM	dry matter
TP	total protein
ALB	albumin
BUN	blood urea nitrogen
GLU	glucose
ALT	alanine aminotransferase
AST	aspartate aminotransferase
T-AOC	total antioxidant capacity
SOD	superoxide dismutase
MDA	malondialdehyde
CAT	catalase
GSH-Px	glutathione peroxidase
IgA	immunoglobulin A
IgG	immunoglobulin G
IL-4	interleukin-4
IL-6	interleukin-6
TNF-α	tumor necrosis factor-α
ELISA	enzyme-linked immunosorbent assay
VIP	variable influence on projection
PCA	principal component analysis
PLS-DA	partial least-squares discriminant analysis
OPLS-DA	orthogonal partial least-squares discriminant analysis

## References

[1] Zhang S., Sun D. (2021). Yesterday, Today and Tomorrow of Dairy Cattle Breeding. China Anim. Ind..

[2] Fu Y., Wang J., Lu Y.Q., Qi Z.G., Guo J.P., Li S.L. (2021). Effects of Feeding Pattern on Growth Performance, Body Size Indices and Serological Indices of Montbeliard × Holstein Crossed Calves. Chin. J. Anim. Nutr..

[3] Wang Z.G., Chang Y., Qiu X.T., Sun Z.H., Zhang B.S., Wang Y.C. (2017). The Analysis of Efficiency of Crossbred Cows between German Simmental and Holstein. China Dairy Cattle.

[4] Pipino D.F., Piccardi M., Lopez-Villalobos N., Hickson R.E., Vázquez M.I. (2023). Fertility and survival of Swedish Red and White? Holstein crossbred cows and purebred Holstein cows. J. Dairy Sci..

[5] Sun S.H., He S.J., Sun Z.Y., Kong L.N., Zhu B., Cao X. (2016). Comparative study on milk performance, fertility and mastitis resistance between Holstein crossing heifers and Holstein heifers. Chin. J. Vet. Sci..

[6] Knob D.A., Scholz A.M., Moro Alessio D.R., Bergamaschi Mendes B.P., Perazzoli L., Kappes R., Neto A.T. (2020). Reproductive and productive performance, udder health, and conformation traits of purebred Holstein, F1, and R1 crossbred Holstein x Simmental cows. Trop. Anim. Health Prod..

[7] Lu N., Zhao K., Xu B., Chen L., Yao D., Chen L., Ma Y. (2023). Comparative Study on Blood Physiology, Biochemistry and Milk Quality between Holstein and Fleckvieh × Holstein F1 Lactating Cow. J. Domest. Anim. Ecol..

[8] Heins B.J., Hansen L.B., Seykora A.J. (2006). Fertility and survival of pure Holsteins versus crossbreds of Holstein with Normande, Montbeliarde, and Scandinavian Red. J. Dairy Sci..

[9] Blöttner S., Heins B.J., Wensch-Dorendorf M., Hansen L.B., Swalve H.H. (2011). Brown Swiss x Holstein crossbreds compared with pure Holsteins for calving traits, body weight, backfat thickness, fertility, and body measurements. J. Dairy Sci..

[10] Chang H., Wang X., Zeng H., Zhai Y., Huang N., Wang C., Han Z. (2023). Comparison of ruminal microbiota, metabolomics, and milk performance between MontbeliardexHolstein and Holstein cattle. Front. Vet. Sci..

[11] Yuan L.G., Liu W., Wang T. (2018). Comparative Study on Growth Performance of the Hybrid Generation between Montbeliard and Holstein. China Cattle Sci..

[12] Kuczyńska B., Puppel K., Gołębiewski M., Kordyasz M., Grodzki H., Brzozowski P. (2012). Comparion of fat and protein fractions of milk constituents in Montbeliarde and Polish Holstein-Friesian cows from one farm in Poland. Acta Vet Brno.

[13] Ma Z., Qiao L., Cong H.M., Sha L.J., Fang Y.J., Zhu Z.Y., Zhong D.B., Liu L., Zhang S.L. (2013). Study on Grouth and Development of the Montbeliarde and Holstein Cattle in Northern Region of China. China Dairy Cattle.

[14] Zhang X., Zhang X., Jiao S., Yu F., Ge J., Zhao J., Wang Y. (2019). Study on Comparisons of Early Growth and Development Performance between Montbeliarde Crossbred and Purebred Holstein. China Dairy Cattle.

[15] Stringer K.A., McKay R.T., Karnovsky A., Quémerais B., Lacy P. (2016). Metabolomics and its Application to Acute Lung Diseases. Front. Immunol..

[16] Qin N., Qin M., Shi W., Kong L., Wang L., Xu G., Guo Y., Zhang J., Ma Q. (2022). Investigation of pathogenesis of hyperuricemia based on untargeted and targeted metabolomics. Sci. Rep..

[17] Ghatak A., Chaturvedi P., Weckwerth W., Varshney R.K., Pandey M.K., Chitikineni A. (2018). Metabolomics in Plant Stress Physiology. Plant Genetics and Molecular Biology.

[18] Xue M.-Y., Sun H.-Z., Wu X.-H., Liu J.-X., Guan L.L. (2020). Multi-omics reveals that the rumen microbiome and its metabolome together with the host metabolome contribute to individualized dairy cow performance. Microbiome.

[19] Zhang J., Gaowa N., Wang Y., Li H., Cao Z., Yang H., Zhang X., Li S. (2023). Complementary hepatic metabolomics and proteomics reveal the adaptive mechanisms of dairy cows to the transition period. J. Dairy Sci..

[20] Valerio A., Casadei L., Giuliani A., Valerio M. (2020). Fecal Metabolomics as a Novel Noninvasive Method for Short-Term Stress Monitoring in Beef Cattle. J. Proteome Res..

[21] Boudra H., Noziere P., Cantalapiedra-Hijar G., Traikia M., Martin J.F., Petera M., Lagree M., Doreau M., Morgavi D.P. (2022). Spot urine collection: A valid alternative to total urine collection for metabolomic studies in dairy cattle. J. Dairy Sci..

[22] Li L., Li F., Hu X., Wu Z., Ren W., Wang T., Ji Z., Li N., Gu J., Sun C. (2022). LAP3 contributes to IFN-γ-induced arginine depletion and malignant transformation of bovine mammary epithelial cells. BMC Cancer.

[23] Humer E., Kroeger I., Neubauer V., Reisinger N., Zebeli Q. (2019). Supplementation of a clay mineral-based product modulates plasma metabolomic profile and liver enzymes in cattle fed grain-rich diets. Animal.

[24] Gomez E., Salvetti P., Gatien J., Carrocera S., Martin-Gonzalez D., Munoz M. (2020). Blood Plasma Metabolomics Predicts Pregnancy in Holstein Cattle Transferred with Fresh and Vitrified/Warmed Embryos Produced in Vitro. J. Proteome Res..

[25] Karisa B.K., Thomson J., Wang Z., Li C., Montanholi Y.R., Miller S.P., Moore S.S., Plastow G.S. (2014). Plasma metabolites associated with residual feed intake and other productivity performance traits in beef cattle. Livest. Sci..

[26] Foroutan A., Fitzsimmons C., Mandal R., Berjanskii M.V., Wishart D.S. (2020). Serum Metabolite Biomarkers for Predicting Residual Feed Intake (RFI) of Young Angus Bulls. Metabolites.

[27] Ilves A., Harzia H., Ling K., Ots M., Soomets U., Kilk K. (2012). Alterations in milk and blood metabolomes during the first months of lactation in dairy cows. J. Dairy Sci..

[28] Demurtas A., Pescina S., Nicoli S., Santi P., Ribeiro de Araujo D., Padula C. (2021). Validation of a HPLC-UV method for the quantification of budesonide in skin layers. J. Chromatogr. B Anal. Technol. Biomed Life Sci..

[29] Zelena E., Dunn W.B., Broadhurst D., Francis-McIntyre S., Carroll K.M., Begley P., O’Hagan S., Knowles J.D., Halsall A., Consortium H. (2009). Development of a robust and repeatable UPLC-MS method for the long-term metabolomic study of human serum. Anal. Chem..

[30] Want E.J., Masson P., Michopoulos F., Wilson I.D., Theodoridis G., Plumb R.S., Shockcor J., Loftus N., Holmes E., Nicholson J.K. (2013). Global metabolic profiling of animal and human tissues via UPLC-MS. Nat. Protoc..

[31] Smith C.A., Want E.J., O’Maille G., Abagyan R., Siuzdak G. (2006). XCMS: Processing mass spectrometry data for metabolite profiling using Nonlinear peak alignment, matching, and identification. Anal. Chem..

[32] Navarro-Reig M., Jaumot J., Garcia-Reiriz A., Tauler R. (2015). Evaluation of changes induced in rice metabolome by Cd and Cu exposure using LC-MS with XCMS and MCR-ALS data analysis strategies. Anal. Bioanal. Chem..

[33] Thevenot E.A., Roux A., Xu Y., Ezan E., Junot C. (2015). Analysis of the Human Adult Urinary Metabolome Variations with Age, Body Mass Index, and Gender by Implementing a Comprehensive Workflow for Univariate and OPLS Statistical Analyses. J. Proteome Res..

[34] Chwalibog A., Tauson A.H., Thorbek G. (2004). Energy metabolism and substrate oxidation in pigs during feeding, starvation and re-feeding. J. Anim. Physiol. Anim. Nutr..

[35] Zhu X.L., Liu W.X., Chen H. (2014). Effect of Thyme Essential Oil on Growth Performance, Serum Protein and Cytokine of Mahua Broilers. China Anim. Husb. Vet. Med..

[36] Wang Y., Zheng X.Y., Gu Q.H., Zheng W.J., Lin B., Shen J.S. (2024). Comparative study on nutrient digestion, rumen fermentation and blood biochemical indexes of water buffalo bulls and Holstein bulls. Anim. Husb. Vet. Med..

[37] Regmi N., Wang T., Crenshaw M.A., Rude B.J., Liao S.F. (2018). Effects of dietary lysine levels on the concentrations of selected nutrient metabolites in blood plasma of late-stage finishing pigs. J. Anim. Physiol. Anim. Nutr..

[38] Liu H.W., Zhou D.W., Li K. (2013). Effects of chestnut tannins on performance and antioxidative status of transition dairy cows. J. Dairy Sci..

[39] Kala M., Shaikh M.V., Nivsarkar M. (2017). Equilibrium between anti-oxidants and reactive oxygen species: A requisite for oocyte development and maturation. Reprod. Med. Biol..

[40] Jaeschke H. (1995). Mechanisms of oxidant stress-induced acute tissue injury. Proc. Soc. Exp. Biol. Med..

[41] Upadhyayula P.S., Higgins D.M., Mela A., Banu M., Dovas A., Zandkarimi F., Patel P., Mahajan A., Humala N., Nguyen T.T.T. (2023). Dietary restriction of cysteine and methionine sensitizes gliomas to ferroptosis and induces alterations in energetic metabolism. Nat. Commun..

[42] Yue X., Feng Q., Zhang H. (2007). Influence of Bovine Unit Distinction to Blood Serum IgG. J. Shenyang Agric. Univ..

[43] Kong F., Zhang S., Liu M., Zhu M., Zhu Y., Zhang C., Li Y. (2019). Comparative Analysis of Blood Physiological and Biochemical Indices between German Simmental Cattle and Holstein Cattle and Their Hybrid Progenies. China Herbiv. Sci..

[44] Zhao P.-F., Wu Y., Li X.-R., Huangfu M.-K., Chen Z.-M., Chen H., Simujide, Wang C.-J., Li C.-J., Bao H. (2023). Differences in Protein Merabolism, Immune Function and Antioxidant Capacity of Chinese Simmental Cattle and Their Hybrid Cattle. Chin. J. Anim. Nutr..

[45] Yang L., Wang Y., Li S., Zhu M., He K., Yao X., Zhang L. (2018). Differential expression of interferon-gamma, IL-4 and IL-10 in peripheral blood mononuclear cells during early pregnancy of the bovine. Reprod. Biol..

[46] Zhu L.J. (2010). Preparation of Monoclonal Antibody to Porcine TNF-α and Expression of TNF-α in PAM Infected with PRRSV.

[47] Zhang B., Zeng M., Wang Y., Li M., Wu Y., Xu R., Zhang Q., Jia J., Huang Y., Zheng X. (2022). Oleic acid alleviates LPS-induced acute kidney injury by restraining inflammation and oxidative stress via the Ras/MAPKs/PPAR-γ signaling pathway. Phytomedicine.

[48] Ho I.C., Miaw S.C., Ma X. (2016). Regulation of IL-4 Expression in Immunity and Diseases. Regulation of Cytokine Gene Expression in Immunity and Diseases.

[49] Yao W.L., Zhang L., Hua Y.L., Ji P., Li P.L., Li J.X., Zhong L.J., Zhao H.F., Wei Y.M. (2015). The investigation of anti-inflammatory activity of volatile oil of *Angelica sinensis* by plasma metabolomics approach. Int. Immunopharmacol..

[50] Danliets M.G., Bel’skii Y.P., Bel’skaya N.V., Trofimova E.S., Uchasova E.G., Ligacheva A.A., Agafonov V.I. (2008). Antiallergic effect of D-glucuronic acid. Eksperimental’naya I Klin. Farmakol..

[51] Smirnoff N. (2001). L-ascorbic acid biosynthesis. Vitamins and Hormones. Cofactor Biosynthesis: A Mechanistic Perspective.

[52] Kuang W.Y., Yang J.J., Liu Z.Y., Zeng J.Z., Xia X.W., Chen X.D., Zhong S.Y., Huang R.M. (2022). Catechin Mediates Ferroptosis to Exert an Anti-Inflammatory Effect on RAW 264.7 Cells. Foods.

[53] Liedhegner E.A.S., Gao X.H., Mieyal J.J. (2012). Mechanisms of Altered Redox Regulation in Neurodegenerative Diseases-Focus on S-Glutathionylation. Antioxid. Redox Signal..

[54] Brosnan J.T., Brosnan M.E. (2006). The sulfur-containing amino acids: An overview. J. Nutr..

[55] Linos A., Kaklamanis E., Kontomerkos A., Koumantaki Y., Gazi S., Vaiopoulos G., Tsokos G.C., Kaklamanis P. (1991). The effect of olive oil and fish consumption on rheumatoid arthritis--a case control study. Scand. J. Rheumatol..

[56] Solanas M., Hurtado A., Costa I., Moral R., Menendez J.A., Colomer R., Escrich E. (2002). Effects of a high olive oil diet on the clinical behavior and histopathological features of rat DMBA-induced mammary tumors compared with a high corn oil diet. Int. J. Oncol..

[57] Bhattacharjee B., Pal P.K., Chattopadhyay A., Bandyopadhyay D. (2020). Oleic acid protects against cadmium induced cardiac and hepatic tissue injury in male Wistar rats: A mechanistic study. Life Sci..

[58] Santa-María C., López-Enríquez S., Montserrat-de la Paz S., Geniz I., Reyes-Quiroz M.E., Moreno M., Palomares F., Sobrino F., Alba G. (2023). Update on Anti-Inflammatory Molecular Mechanisms Induced by Oleic Acid. Nutrients.

[59] Hong J.H., Lee Y.C. (2022). Anti-Inflammatory Effects of Cicadidae Periostracum Extract and Oleic Acid through Inhibiting Inflammatory Chemokines Using PCR Arrays in LPS-Induced Lung inflammation In Vitro. Life.

